# MiR-17-92 cluster is associated with 13q gain and c-myc expression during colorectal adenoma to adenocarcinoma progression

**DOI:** 10.1038/sj.bjc.6605037

**Published:** 2009-08-11

**Authors:** B Diosdado, M A van de Wiel, J S Terhaar Sive Droste, S Mongera, C Postma, W J H J Meijerink, B Carvalho, G A Meijer

**Affiliations:** 1 Department of Pathology, VU University medical center Amsterdam, Amsterdam 1081HV, the Netherlands; 2 Department of Biostatistics, VU University medical center Amsterdam, Amsterdam 1081HV, the Netherlands; 3 Department of Mathematics, VU University medical center Amsterdam, Amsterdam 1081HV, the Netherlands; 4 Department of Gastroenterology, VU University medical center Amsterdam, Amsterdam 1081HV, the Netherlands; 5 Department of Surgery, VU University medical center Amsterdam, Amsterdam 1081HV, the Netherlands

**Keywords:** colorectal adenoma to carcinoma progression, DNA copy number changes, miRNA-17-92 cluster expression

## Abstract

**Background:**

MicroRNAs are small non-coding RNA molecules, which regulate central mechanisms of tumorigenesis. In colorectal tumours, the combination of gain of 8q and 13q is one of the major factors associated with colorectal adenoma to adenocarcinoma progression. Functional studies on the miR-17-92 cluster localised on 13q31 have shown that its transcription is activated by c-myc, located on 8q, and that it has oncogenic activities. We investigated the contribution of the miR-17-92 cluster during colorectal adenoma to adenocarcinoma progression.

**Methods:**

Expression levels of the miR-17-92 cluster were determined in 55 colorectal tumours and in 10 controls by real-time RT–PCR. Messenger RNA c-myc expression was also determined by real-time RT–PCR in 48 tumours with array comparative genomic hybridisation (aCGH) data available.

**Results:**

From the six members of the miR-17-92 cluster, all except miR-18a, showed significant increased expression in colorectal tumours with miR-17-92 locus gain compared with tumours without miR-17-92 locus gain. Unsupervised cluster analysis clustered the tumours based on the presence of miR-17-92 locus gain. Significant correlation between the expression of c-myc and the six miRNAs was also found.

**Conclusion:**

Increased expression of miR-17-92 cluster during colorectal adenoma to adenocarcinoma progression is associated to DNA copy number gain of miR17-92 locus on 13q31 and c-myc expression.

MicroRNAs (miRNAs), which are small non-coding RNA molecules of 18–25 nucleotides long, have been shown to have an important role in cancer. They function by downregulating the expression of multiple target genes by degrading the mRNA or blocking their translation into proteins (Bartel, 2004; Esquela-Kerscher and Slack, 2006). These molecules regulate genes involved in central pathways to cellular homoestasis, development and tumorigenesis, including proliferation, apoptosis, differentiation, maintenance of stem cell potency and angiogenesis (Lee *et al*, 1993; Wightman *et al*, 1993; Reinhart *et al*, 2000; Brennecke *et al*, 2003; Chen *et al*, 2004; Zhao *et al*, 2007). Specific miRNA expression signatures have been described in different cancer (sub)types, and within these cancer types, different miRNA expression signatures predict clinical outcome (Calin and Croce, 2006). In several instances, expression of miRNAs was shown to have a function in tumour biology. One of the first studies published described the tumour suppressor activity of let-7 in lung cancers by downregulating the *Ras* oncogene (Johnson *et al*, 2005). Yet, the cause of disturbed miRNA expression in cancer has only partially been elucidated. MiRNAs appear to be frequently located on regions of genomic instability (Calin *et al*, 2004) and miRNA expression changes have been found to be associated with chromosomal rearrangements in ovarian cancer, breast cancer, lung cancer, melanomas and hematopoietic malignancies (Calin *et al*, 2002; Hayashita *et al*, 2005; Lu *et al*, 2005; Zhang *et al*, 2006; Tagawa *et al*, 2007). Alternatively, epigenetic regulation of miRNA expression has been described in colorectal, breast, lung cancers and different cancer cell lines (Saito *et al*, 2006; Lujambio *et al*, 2007).

The miR-17-92 cluster (oncomir-1), located within the third intron of the open reading frame 13 (*C13orf25*), encompasses six miRNAs (miR-17, miR-18a, miR-19a, miR-20a, miR-19b-1 and miR-92a-1) over ∼800 nucleotides on 13q31.3. Amplification of this locus and overexpression of the miR-17-92 cluster have been documented in B-cell lymphomas and lung cancer (Hayashita *et al*, 2005; Rinaldi *et al*, 2007). The transcription factors c-myc (8q24) and E2F3 induce the expression of the miR-17-92 cluster. These observations are consistent with a complex regulatory network where apoptotic and proliferative signals of c-myc and E2F1 are tightly balanced by the expression of the miR-17-92 cluster (O'Donnell *et al*, 2005; Sylvestre *et al*, 2007; Woods *et al*, 2007).

Colorectal cancer (CRC) is a heterogeneous disease, which develops through the accumulation of multiple (epi)genetic alterations. Accepted models of CRC progression describe mutations in the Wnt pathway as an initiation event that leads to adenoma formation (Fearon and Vogelstein, 1990). A key step in subsequent progression to cancer is genomic instability, that occurs in about 5% of adenomas, either through failing DNA mismatch repair (ca. 0.5%, corresponding to 15% of all sporadic CRCs) giving rise to the mutator phenotype marked by microsatellite instability (MSI), or (ca. 4.5%, corresponding to 85% of all sporadic CRCs) (increased levels of) chromosomal instability (CIN). The latter gives rise to aneuploid tumours showing a non-random pattern of chromosomal alterations frequently, including 8q, 13q and 20q gains and 8p, 15q, 17p and 18q losses (Hermsen *et al*, 2002). Several studies have shown gene dosage effects on mRNA expression of genes in these gained or lost chromosomal areas (Platzer *et al*, 2002). Given the gains of 13q, which includes the miR-17-92 cluster, and gain of 8q, including frequent c-myc amplification, led us to test the hypothesis that gene dosage effects of the miR-17-92 cluster are associated with colorectal adenoma to adenocarcinoma progression. Our results indicated that miR-17-92 increased expression during colorectal adenoma to adenocarcinoma progression is associated to miR-17-92 locus gain and c-myc transcriptional activity.

## Materials and methods

### Patients and tumour tissues

Fresh tissue samples from 55 patients with colorectal tumours (30 adenomas and 25 adenocarcinomas) were collected prospectively at the department of pathology of the VU – University medical center Amsterdam (VUmc) – Amsterdam, the Netherlands. In addition, normal colorectal epithelium was obtained by brushing the normal mucosa of colon resection margins of 10 individuals (mean age 68 years) who underwent a colon resection. All 10 colon resection margins were located at least 10 cm from the tumour and histologically classified as cancer-free. Clinicopathological characteristics are listed in Table 1. Array comparative genomic hybridisation (aCGH) data collected using BAC arrays printed in house, containing ∼5000 DNA clones with an average resolution along the whole genome of 1.0 Mb, including contig coverage of 13q, (http://www.vumc.nl/microarrays/index.html) were available from the same sample of 48 patients with colorectal tumours (GEO accession number GSE8067). Of these same samples, total RNA was isolated using TRIzol (Invitrogen, Carlsbad, CA, USA) following the manufacturer's guidelines with some modifications (http://www.vumc.nl/microarrays/index.html). Total RNA quantity was determined with a Nanodrop ND-1000 spectrophotometer (Isogen, Hackensack, NJ, USA) and quality was assessed in a 1% agarose gel, stained with ethidium bromide. The study was carried out in accordance with the ethical guidelines of the VU Medical Center of Amsterdam, concerning the use of leftover patient material.

### Quantitative RT–PCR and real-time TaqMan PCR

Quantification of *c-myc* mRNA expression levels was carried out by real-time RT–PCR using SYBR Green (Applied Biosystems, Foster City, CA, USA). First, 2 *μ*g of total RNA were incubated at 37°C with 2 *μ*l of DNase I (1000 U) (Invitrogen), 1 *μ*l of 10 × buffer and 0.5 *μ*l of Rnasin (2.500 U; Promega, Madison, WI, USA) in a final volume of 10 *μ*l. After 30′, the reaction was stopped by adding 1 *μ*l of DNAse stop solution and incubating at 65°C for 10′. Second, RT reactions were conducted using 1 *μ*g of DNAse-treated total RNA as starting material, which was incubated for 10′ at 70°C with 1 *μ*l of oligo(dT)_20_ Primer (Invitrogen) in a final volume of 6 *μ*l. Next, cDNA synthesis was completed by adding 0.25 *μ*l of Superscript II reverse transcriptase (300 U; Invitrogen), 2 *μ*l of 10 × RT-buffer, 10 *μ*l of dNTPs (2 mM), 2 *μ*l DTT (10 mM) and 0.25 *μ*l of RNasin (2500 U) in a final volume of 21 *μ*l, during 60′ at 42°C and at 95°C for 5′. Complementary DNA was diluted with DEPC-treated water in a final volume of 100 *μ*l and stored at −20°C until used. Last, RT–PCR was carried out using primers directed to *c-myc* (Fwd: 5′ CAG CTG CTT AGA CGC TGG ATT 3′, Rev: 5′ GTA GAA ATA CGG CTG CAC CGA 3′ with an annealing temperature (Ta) of 60°C) and the housekeeping gene *β2M* (Fwd: 5′ TGA CTT TGT CAC AGC CCA AGA TA 3′ and Rev: 5′ AAT GCG GCA TCT TCA AAC CT 3′ with a Ta of 57°C). For each reaction, 25 ng of cDNA was used as starting material and a master mix containing 12.5 *μ*l of SYBR Green PCR master mix (Applied Biosystems) and 1.25 *μ*l of each of the primers sets until a final volume of 25 *μ*l. Reactions were conducted in duplo in a 7300 Real-time PCR System (Applied Biosystems). Amplification conditions comprised a denaturation step 10′ at 95°C and 50 cycles at 95°C for 15 ″ and gene-specific annealing temperature for 1′.

To determine the expression levels of the MiR-17-92 cluster genes, six Taqman microRNA assays (Applied Biosystems) directed to hsa-miR-17 (ABI 4373119), hsa-miR-18a (ABI 4373118), hsa-miR-19a (ABI 4373099), hsa-miR-20a (ABI 4373286), hsa-miR-19b-1 (ABI 4373098), hsa-miR-92a-1 (ABI 4373013) and an endogenous reference, the *RNU48* gene (ABI 4373383), were used following the manufacture's protocol using 10 ng of total RNA as input material. All reactions were carried out in duplo in a 7300 Real-time PCR System (Applied Biosystems).

### Statistical analysis

The expression levels of *c-myc* and the miR-17-92 cluster were calculated from the obtained *C*_t_ values using the Δ *C*_t_ method as described previously (Livak and Schmittgen, 2001). Box and scatter plots were used to appreciate the descriptive statistics of the data. Correlation coefficients between the expression levels of the miR-17-92 cluster genes were obtained by Spearman correlation (SPSS 14.0 for Windows, SPSS Inc., Chicago, IL, USA). Significance of differences in expression levels between tumours with and without miR-17-92 locus gain was computed by the Mann–Whitney *U* non-parametric test for independent samples (SPSS 14.0 for Windows). A multivariate analysis of the association of the expression of the miR-17-92 cluster with miR-17-92 locus gain, accounting for correlation between the six miRs in the cluster, was carried out using a linear mixed effect model in combination with an ANOVA *F*-test (‘nlme’ library R, http://cran.r-project.org/). Unsupervised cluster analysis was carried out using the parameters complete linkage and Euclidean distance (R software version 2.6.1). The Pearson *χ*^2^-test was used for analysing associations between cluster membership and presence or absences of miR-17-92 locus DNA copy number aberrations (SPSS 14.0 for Windows).

## Results

### The MiR-17-92 cluster is overexpressed during colorectal adenoma to adenocarcinoma progression

**Figure 1:**
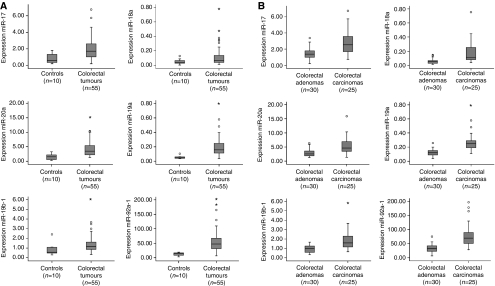
Expression levels of miR-17-92 cluster in 55 colorectal tumours (30 colorectal adenomas and 25 adenocarcinomas) and 10 controls. (**A**) Box plots with median, 25th and 75th percentiles and range of expression levels of each of the six components of the miR-17-92 cluster in 10 normal colon epithelium samples and 55 colorectal tumours showed significantly increased expression in colorectal tumours compared with the controls. (**B**) Box plots with median, 25th and 75th percentiles and range of expression levels of each of the six components of the miR-17-92 cluster in 30 colorectal adenomas and 25 adenocarcinomas showed significantly increased expression in colorectal adenocarcinomas compared with the adenomas.

To reveal the role of the miR-17-92 cluster in CRC pathogenesis, the expression levels of the six members of this cluster (miR-17, miR-18a, miR-19a, miR-20a, miR-19b-1 and miR-92a-1) were determined by real-time RT–PCR in 55 colorectal tumours (30 adenomas and 25 adenocarcinomas) and 10 controls (normal colorectal epithelium). The expression levels of miR-17 showed 2.6-fold increase in CRC tumours compared with controls (*P*=0.001), as well as for miR-18a, which displayed 2.4-fold increase (*P*=0.04), miR-19a with 3.4-fold increase (*P*<0.001), miR-20a with 2.6 elevated expression (*P*=0.001), miR-19b-1 with 1.6-fold changes (*P*=0.021) and miR-92a-1 with 4.5 increased expression (*P*<0.001; Figure 1A).

Next, to study whether these miRNAs play a role during colorectal adenoma to adenocarcinoma progression, we also analysed differences in expression between adenomas and adenocarcinomas. Indeed, all six miRNAs showed higher expression in adenocarcinomas than in adenomas, with miR-17 expression being 1.9-fold (*P*<0.001) increased, miR-18a 3.4-fold (*P*<0.001), miR-19a, 2.3-fold (*P*<0.001), miR-20a, 2.0-fold (*P*<0.001), miR-19b-1, 2.0-fold (*P*<0.001) and miR-92a-1, 2.4-fold increased (*P*<0.001) (Figure 1B). These observations indicate a role of miRNAs during colorectal adenoma to adenocarcinoma progression.

### Overexpression of MiRNA cluster 17-92 members is significantly associated with DNA copy number gain of the miR-17-92 locus on 13q31

**Figure 2:**
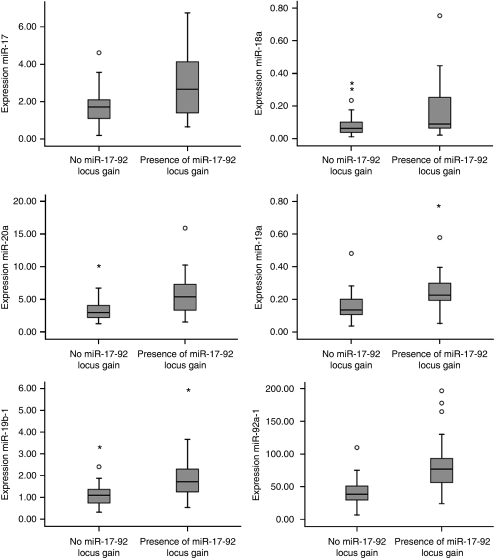
Expression levels of the six miRNA-17-92 cluster members by DNA copy number status of the miR-17-92 locus in 48 colorectal tumours. Box plots with median, 25th and 75th percentiles and range of expression levels of each of the six miRNAs of the miR-17-92 cluster in 48 colorectal tumours showed significantly increased expression in colorectal tumours with miR-17-92 locus gain compared with colorectal tumours without gain of the miR-17-92 locus except miR-18a.

To study whether DNA copy number gain of miR-17-92 locus in colorectal tumours influences the expression of the miR-17-92 cluster, 48 of the set of 55 colorectal tumours, with aCGH data available were selected. Seventeen tumours (4 adenomas and 13 adenocarcinomas) showed 13q DNA copy number gain and 31 tumours (21 adenomas and 10 adenocarcinomas) did not show gain of the miR-17-92 locus. Comparison of the expression levels of each of the miR-17-92 cluster miRNAs between these two groups showed that expression of miR-17 was increased 1.6-fold (*P*=0.05) in tumours with miR17-92 locus gain compared with tumours with no miR-17-92 locus gain. In addition, miR-19a showed 1.8 times elevated expression (*P*<0.01), miR-20a 1.7-fold (*P*<0.01), miR-19b-1 1.8-fold (*P*<0.003) and miR-92a-1 2.0 times increased expression (*P*<0.003). MiR-18a showed a 2.0-fold over expression that did not reach statistical significance (*P*=0.29) (Figure 2). The multivariate analysis for associating expression values of the entire miR cluster with miR17-92 locus gain revealed a highly significant association (*P*<0.001).

**Figure 3:**
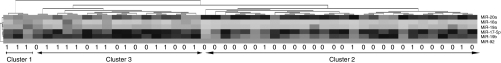
Hierarchical cluster analysis of 48 colorectal tumours based on miR-17-92 cluster expression levels. Unsupervised cluster analysis of the expression levels of the six members of the miR-17-92 cluster across the 48 colorectal tumour groups separate the colorectal tumours with gain of the miR-17-92 locus (clusters 1 and 3) from the tumours with no gain at this locus (cluster 2). Each column represents a colorectal tumour and each row represents the expression of each of the components of the miR-17-92 cluster. Zero (0) represent colorectal tumours with no miR-17-92 locus gain and 1 represent colorectal tumours with miR-17-92 locus gain.

Unsupervised hierarchical cluster analysis of the 48 tumours with aCGH data available, revealed three clusters on the basis of the expression levels of these six miRNAs (Figure 3). Significant association between cluster membership and DNA copy number status of the six miRNAs was found (*P*=0.001). Cluster 1 grouped three of samples with gain of miR17-92 locus. Cluster 2 comprised only four of the samples with gain of miR17-92 locus and 24 of the samples with no miR17-92 locus gain, whereas cluster 3 contained 10 of the samples with miR17-92 locus gain and seven of the tumours with no miR17-92 locus gain (Table 2).

In conclusion, these data indicate a gene dosage effect of miR17-92 locus amplification on expression levels of miRNA at this locus during colorectal adenoma to adenocarcinoma progression.

### Expression of the miR-17-92 cluster correlates with c-myc expression in colorectal tumours

**Figure 4:**
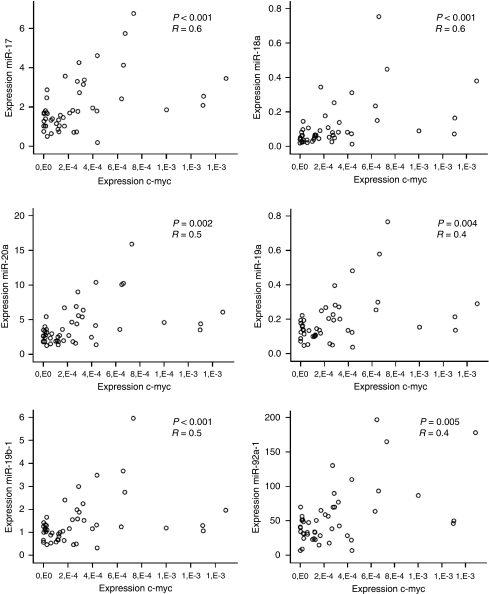
Correlation between the expression of the *c-myc* gene and each of the miRNAs of the miR-17-92 cluster. The scatter plots of c-myc mRNA expression (x axis) and expression of each of the miR-17-92 cluster miRNAs (y axis) show positive correlations.

MiR-17-92 expression has also been shown to be regulated by the transcription factor c-myc, located on 8q24. Consequently, to fully investigate the mechanisms of regulation of the expression of the miR-17-92 cluster during colorectal adenoma to adenocarcinoma progression, we determined the correlation between the *c-myc* mRNA expression on 48 tumours with miR-17-92 expression data available. *c-myc* expression levels were significantly correlated to the expression of miR17-5p (*r*=0.6, *P*<0.001), miR-18a (*r*=0.6, *P*<0.001), miR-20a (*r*=0.5, *P*<0.001), miR-19a (*r*=0.5, *P*=0.002), miR-19b-1 (*r*=0.4, *P*=0.004) and miR-92a-1 (*r*=0.4, *P*<0.005) as shown in Figure 4.

The correlation between *c-myc* and miR-17-92 expression illustrates the transcriptional regulation of c-myc on the miR-17-92 cluster in CRC tumours.

## Discussion

Onset or substantial increase in level of CIN, depending on the definition, is a major pathogenetic mechanism in colorectal adenoma to adenocarcinoma progression. Presence of 13q gain is one of the major factors associated with colorectal adenoma to adenocarcinoma progression in CIN tumours (Hermsen *et al*, 2002). MicroRNAs contribute to tumorigenesis as tumour suppressor genes or oncogenes due to their altered expression. An effect of DNA copy number variations implicated in human cancers on miRNAs expression has been documented previously (Calin and Croce, 2006). The region of chromosomal gain on 13q harbours the miR-17-92 cluster at locus 13q31.3. The six components of this cluster are located in the third intron of the *C13orf25* gene and span nearly 800 bases. The *C13orf25* gene encodes for a protein of 70 amino acids with no putative domains and unknown function, and is mainly considered to be a carrier of the miR-17-92 cluster (Mendell, 2008). Studies in lung cancer have shown that its transcripts are mainly localised in the nucleus, suggesting that translation of the mRNA into protein is limited. Functional experiments have shown that whereas transfection of the miRNAs of the miR-17-92 cluster into lung cells lead to increased proliferation, transfection of expression constructs containing C13orf25 cDNA did not lead to any phenotypic change in the same cells (Hayashita *et al*, 2005). Interestingly, the conservation of the exons of the *C13orf25* among species is low, compared with the miR-17-92 cluster, which is conserved in all vertebrates (Mendell, 2008).

In this study, we described that DNA copy number gain of the miR-17-92 locus was associated with increased expression of all components of the miR-17-92 cluster except miR-18a. Lack of correlation in increased expression between miR-18a and the other members of the miR-17-92 cluster has been found when K562 leukaemia cells were transfected with a miR-17-19b construct (Venturini *et al*, 2007). In this study, the influence of DNA copy number gain of the miR-17-92 locus during CRC progression was further supported by an unsupervised cluster analysis. This analysis showed that the expression levels of the six miRNAs segregated with miR-17-92 locus gain in the colorectal tumours studied. Furthermore, the miR-17-92 cluster, located on 13q31, has previously been described as being regulated by the transcription factor c-myc, located on 8q24 (Chang *et al*, 2008). Indeed, a positive correlation was found between c-myc expression and expression of each of the members of the miRNA cluster in this study, indicating the influence of c-myc transcriptional activity on the miR-17-92 during CRC progression.

Expression profiling studies comparing controls with colorectal adenocarcinomas had already shown increased expression of some members of this cluster (Bandres *et al*, 2006; Cummins *et al*, 2006; Volinia *et al*, 2006). However, these studies used whole normal mucosa biopsies as source of RNA, including the mixture of non-epithelial cells that populate the lamina propria. In this study, we have isolated samples containing as pure a population of normal epithelial cells as possible by brushing the surface epithelium of the colorectal mucosa in resection specimens. This approach has previously been validated by making cytological specimens of these samples, and showed a high concentration of epithelial cells (Meijer *et al*, 1998). Therefore, our results indicate more accurately that differences in expression of the miRNA miR-17-92 cluster observed between controls and colorectal carcinomas actually reflect differences in the biology of normal and neoplastic epithelial cells. Yet, the cause of this altered expression had not been studied, nor was their role in colorectal adenoma to adenocarcinoma progression. Functional studies on B-cell lymphomas and mouse colonocytes have shown that overexpression of miR-17-92 on an elevated c-myc background, promotes tumour growth by targeting E2F1 and increased angiogenesis by targeting thrombospondin-1 (*TSP1*) and connective tissue growth factor (*CTGF*; He *et al*, 2005; O'Donnell *et al*, 2005; Dews *et al*, 2006). Other experimentally validated target genes of some of the components of the miR-17-92 cluster include the cyclin-dependednt kinase inhibitor (*CDKN1A*) and the *BCL2-like 11* gene (*BCL2L11*) (Ivanovska *et al*, 2008; Koralov *et al*, 2008; Petrocca *et al*, 2008; Ventura *et al*, 2008; Xiao *et al*, 2008). These genes control cell cycle progression and cell death, respectively; however their participation in CRC tumorigenesis as targets of the miR-17-92 has not been studied. Although the exact mechanisms, that is, the down stream targets, through which the miR-17-92 miRNAs contribute to colorectal adenoma progression have not been identified yet, it is tempting to speculate on the possibility of using antagomiRs to interfere with colorectal adenoma to carcinoma progression (Krutzfeldt *et al*, 2005).

The present data strongly suggest that the increase in CIN that makes colorectal adenomas progress to adenocarcinomas not only leads to upregulation of oncogenes but also causes the overexpresssion of critical miRNAs.

## Figures and Tables

**Table 1:** Clinical and histopathological features of 55 patients and their respective tumours

**Tumour ID**	**Age**	**Gender**	**Tumour type**	**Histology**	**Degree dysplasia**	**Degree differentiation**	**Presence 17–92 locus gain**	**MSI status**
F19C	85	M	Adenocarcinoma	ND	NA	Moderate	No	MSI
F15C	65	F	Adenocarcinoma	ND	NA	Moderate	No	MSI
F33C	83	M	Adenocarcinoma	ND	NA	Moderate	No	MSI
F35C	77	M	Adenocarcinoma	Mucinous	NA	Moderate	No	MSI
F57C	58	F	Adenocarcinoma	ND	NA	Moderate	No	MSI
F39C	88	F	Adenocarcinoma	Mucinous	NA	Moderate	No	MSS
F27C	65	F	Adenocarcinoma	ND	NA	Moderate	No	MSS
F32C	65	F	Adenocarcinoma	ND	NA	Moderate	No	MSS
F8C	80	F	Adenocarcinoma	ND	NA	Poorly	No	MSS
F3C	72	F	Adenocarcinoma	ND	NA	Moderate	No	Unknown
F53C	63	M	Adenocarcinoma	Mucinous	NA	Moderate	Unknown	MSS
F36C	82	M	Adenocarcinoma	ND	NA	Well	Unknown	MSS
F54C	60	F	Adenocarcinoma	ND	NA	Well	Yes	MSS
F11C	66	M	Adenocarcinoma	ND	NA	Moderate	Yes	MSS
F12C	65	M	Adenocarcinoma	ND	NA	Moderate	Yes	MSS
F14C	71	F	Adenocarcinoma	ND	NA	Well	Yes	MSS
F46C	53	M	Adenocarcinoma	mucinous	NA	Moderate	Yes	MSS
F28C	71	F	Adenocarcinoma	ND	NA	Well	Yes	MSS
F30C	74	F	Adenocarcinoma	ND	NA	Poorly	Yes	MSS
F34C	57	F	Adenocarcinoma	ND	NA	Moderate	Yes	MSS
F58C	59	M	Adenocarcinoma	ND	NA	Moderate	Yes	MSS
F51C	47	M	Adenocarcinoma	ND	NA	Well	Yes	MSS
F10C	57	F	Adenocarcinoma	ND	NA	Well	Yes	MSS
F6C	84	F	Adenocarcinoma	ND	NA	Moderate	Yes	MSS
F1C	62	M	Adenocarcinoma	ND	NA	Moderate	Yes	MSS
F7A	70	M	Adenoma	Tubulovillous	Severe	NA	No	MSS
F56A	48	M	Adenoma	Tubular	Moderate	NA	No	MSS
F62A	80	M	Adenoma	Tubular	Moderate	NA	No	MSS
F50A	75	F	Adenoma	Tubulovillous	Moderate	NA	No	MSS
F40A	54	M	Adenoma	Tubular	Moderate	NA	No	MSS
F44A	75	F	Adenoma	Villous	Moderate	NA	No	MSS
F22A	71	F	Adenoma	Tubulovillous	Severe	NA	No	MSS
F23A1	77	M	Adenoma	Tubulovillous	Moderate	NA	No	MSS
F26A	60	M	Adenoma	Tubulovillous	Moderate	NA	No	MSS
F37A	58	M	Adenoma	Tubulovillous	Severe	NA	No	MSS
F38A1	75	M	Adenoma	Tubulovillous	Moderate	NA	No	MSS
F68A	52	M	Adenoma	Tubulovillous	Moderate	NA	No	MSS
F55A	79	F	Adenoma	Tubulovillous (mucinous)	Severe	NA	No	MSS
F58A1	59	M	Adenoma	Tubular	Moderate	NA	No	MSS
F59A	65	F	Adenoma	ND	ND	NA	No	MSS
F61A	70	M	Adenoma	Tubulovillous (serrated)	Severe	NA	No	MSS
F21A	71	F	Adenoma	Tubulovillous	Severe	NA	No	Unknown
F66A	75	M	Adenoma	Tubular	Severe	NA	No	Unknown
F20A	80	M	Adenoma	Tubulovillous	Severe	NA	No	MSS
F47A	62	M	Adenoma	Tubulovillous	Moderate	NA	No	MSS
F38A2	75	M	Adenoma	Tubulovillous	Moderate	NA	No	MSS
F52A	74	F	Adenoma	Tubular	Moderate	NA	Unknown	MSS
F58A2	59	M	Adenoma	Tubular	Moderate	NA	Unknown	MSS
F42A	82	M	Adenoma	Tubular	Moderate	NA	Unknown	MSS
F43A	79	F	Adenoma	Villous	Moderate	NA	Unknown	MSS
F45A	79	F	Adenoma	Villous	Moderate	NA	Unknown	MSS
F48A	72	M	Adenoma	Tubular	Moderate	NA	Yes	MSS
F23A2	77	M	Adenoma	Tubulovillous	Severe	NA	Yes	MSS
F60A	61	M	Adenoma	Tubular	Moderate	NA	Yes	MSS
F64A	55	M	Adenoma	Tubular	Moderate	NA	Yes	MSS

F=female; M=male; MSI=microsatellite instable; MSS=microsatellite stable; NA=not applicable; ND=no data.

**Table 2:** Cluster membership of 48 colorectal tumours based on expression data of the six miRNAs members of the miR-17-92 cluster

	**Tumours with miR17-92 locus gain**	**Tumours with no miR17-92 locus gain**
Cluster 1	3	0
Cluster 2	4	24
Cluster 3	10	7

miRNA=microRNA.
